# The Emerging Role of Sonoelastography in Pregnancy: Applications in Assessing Maternal and Fetal Health

**DOI:** 10.3390/diagnostics15010047

**Published:** 2024-12-28

**Authors:** Abdulrahman M. Alfuraih

**Affiliations:** Radiology and Medical Imaging Department, College of Applied Medical Sciences, Prince Sattam bin Abdulaziz University, Al-Kharj 16278, Saudi Arabia; a.alfuraih@psau.edu.sa

**Keywords:** prenatal diagnosis, sonoelastography, cervical elasticity, fetal tissue assessment, placental stiffness, preterm birth prediction, shear wave elastography, maternal–fetal health, ultrasound imaging, pelvic floor

## Abstract

Sonoelastography, a novel ultrasound-based technique, is emerging as a valuable tool in prenatal diagnostics by quantifying tissue elasticity and stiffness in vivo. This narrative review explores the application of sonoelastography in assessing maternal and fetal health, with a focus on cervical, placental, pelvic floor, and fetal tissue evaluations. In the cervix, sonoelastography aids in predicting preterm birth and assessing labor induction success. For the placenta, it provides insights into conditions like preeclampsia and intrauterine growth restriction through elasticity measurements. Assessing fetal tissues, including the lungs, liver, and brain, sonoelastography offers a non-invasive method for evaluating organ maturity and detecting developmental anomalies. Additionally, pelvic floor assessments enable better management of childbirth-related injuries and postpartum recovery. While current studies support its safety when used within established limits, further research is necessary to confirm long-term effects. Future advancements include refining protocols, integrating machine learning, and combining sonoelastography with other diagnostic methods to enhance its predictive power. Sonoelastography holds promise as an impactful adjunct to conventional ultrasound, providing quantitative insights that can improve maternal and fetal outcomes in prenatal care.

## 1. Introduction

Prenatal diagnosis plays a crucial role in modern obstetric care by enabling the monitoring of maternal and fetal health at various stages of pregnancy. In general, prenatal diagnosis is used in the detection of defects in the fetus and to develop a plan of treatment before or after birth. It also helps in identifying the causes that may be harmful to future pregnancies [1]. Several invasive and non-invasive methods are employed at specific stages in the pregnancy to foresee an exact diagnosis. Ultrasonography has conventionally remained a fundamental, encompassing imaging methodology widely used to visualize the fetus to gain real-time information related to fetal anatomy, growth, and development. Thus far, it is popular for the assessment of morphology and vascularity and lacks the wide use of advanced imaging biomarkers it may provide.

Sonoelastography is one of the new ultrasound-based modalities that measures the stiffness of tissues in vivo. It promises to advance the capabilities of conventional ultrasound by measuring the elasticity or stiffness of tissues through its deformation upon mechanically or acoustically induced pressure [2]. It is based on the pressure force applied to the targeted area and monitors the tissue displacement. The degree of displacement reflects the elasticity or stiffness of the tissue, where softer tissue deforms more easily. These differences are presented as color images or even as quantitative data that also enables clinicians to establish tissues’ biochemical properties.

There are three major types of sonoelastography: shear wave elastography (SWE), strain elastography (SE), and transient elastography (TE) [2]. SWE generates quantitative measurements by evaluating the propagation speed of shear waves induced by focused ultrasound pulses, with faster wave propagation indicating stiffer tissues. These measurements are presented as either wave velocities or Young’s modulus values. SWE is particularly advantageous in assessing deeper tissues, such as the liver or placenta, and in longitudinal studies requiring consistent metrics. However, its performance can be affected by factors such as tissue viscosity and acoustic attenuation, which may compromise accuracy in certain anatomical regions [2].

In contrast, SE assesses tissue stiffness by evaluating deformation in response to an applied mechanical force or due to internal physiologic motion (e.g., cardiovascular, respiratory). This technique is qualitative or semi-quantitative, making it more dependent on operator expertise. While SE excels in assessing superficial tissues, such as the breast or thyroid, its measurements can be variable due to the inherent subjectivity of the operator and variability in applied force [2]. Despite these limitations, SE is relatively cheaper, making it a practical option in settings where advanced SWE systems are unavailable. Meanwhile, TE, a non-imaging method, is employed when a motor vibrates the skin, causing a passing distortion in the tissue, and as that distortion moves deeper into the body, a quantitative one-dimensional image of tissue stiffness is obtained. The TE type is popular in hepatological tissue assessment and is seldom used in gynecological or obstetric assessment.

The role of sonoelastography has been widely established for the assessment of breast, liver, and thyroid. However, the literature on maternal and fetal assessment is relatively smaller, albeit growing. Hence, it is significant to review the status of the clinical applications of sonoelastography to understand and determine the direction for future prenatal diagnostics.

The purpose of this review is to discuss the rapidly increasing use of sonoelastography in prenatal diagnosis and to elucidate its potential role in assessing maternal and fetal health. A structured search was conducted in PubMed, Scopus, and Web of Science databases, using combinations of the keywords: ‘sonoelastography’, ‘shear wave elastography’, ‘strain elastography’, ‘prenatal diagnosis’, ‘cervix’, ‘placenta’, ‘pelvic floor’, and ‘fetal tissue’. Articles published in English between 2010 and 2024 were considered. Current studies have great promise regarding sonoelastography in various fields related to prenatal care, such as uterine and cervical stiffness measurement, abnormal placentas, fetal organs, and soft tissues [3,4,5]. Sonoelastography may sensitize the early detection of complications like preterm labor, placental insufficiency, and fetal growth restrictions due to its ability to detect even slight changes in tissue elasticity. This review sheds light on the elastic properties of various related organs and tissues. It also discusses how this advanced imaging technique may improve maternal and fetal outcomes.

## 2. Cervix Assessment

### 2.1. Cervix Elastic Properties

The cervix plays a very important role in conception, maintaining the pregnancy, and timely delivery of the fetus by acting as a structural barrier that holds the fetus in place within the uterus [6]. It is composed of a complex mixture of smooth muscle fibers, collagen, and extracellular matrix proteins, all of which contribute to its biomechanical properties [7]. During pregnancy, the cervix progresses through four stages, each defined by changes in collagen alignment: (a) an initial softening phase; (b) a phase of shortening and pronounced softening known as ‘ripening’; (c) active dilation; and (d) recovery post-delivery [8]. The initial softening is a gradual process, typically unfolding over days to weeks, while the ripening phase accelerates, occurring over several hours to days. These progressive changes are essential for enabling the fetus’s passage through the cervical canal.

Traditional methods for assessing the cervix, such as internal examination (e.g., Bishop score) and transvaginal ultrasonography, provide limited information on the tissue’s biomechanical properties. While ultrasound can measure cervical length and detect some degree of cervical softening, it does not assess tissue elasticity with high precision. In contrast, sonoelastography offers an advantage by assessing cervical biomechanical changes in real-time.

Recent recommendations by Thomsen et al. for strain elastography emphasize probe placement and region of interest (ROI) selection to enhance measurement reliability [9]. They found that variations in probe-to-ROI distance and angle affected strain values, with posterior cervical assessments yielding consistently lower values due to tissue interference. These guidelines aim to standardize elastography practices, enhancing measurement reliability and thereby improving its clinical relevance for assessing cervical integrity.

Several studies have tracked changes in cervical elasticity and stiffness across gestational ages using elastography, highlighting its potential for monitoring cervical readiness for labor. Fruscalzo et al. demonstrated the reliability and reproducibility of semi-quantitative cervical SE in early and mid-pregnancy, with findings showing an inverse correlation between cervical stiffness and gestational age, particularly strong in the third trimester [10,11]. Hernandez-Andrade et al. observed significant variations in cervical strain across gestational ages, correlating progressive softening with cervical length changes as pregnancy advances [12]. Similarly, Ono et al. noted gestational age-related decreases in SWE stiffness, with the upper cervix proving more sensitive to softening than the lower part [13].

Duan et al. reported a cervical stiffness gradient from the inner to external cervix, with reduced stiffness more evident in multiparous women later in pregnancy [14]. Woo et al. reinforced the suitability of SWE for longitudinal monitoring, particularly at the internal os, which provided consistent readings [15]. Nguyen-Hoang et al. identified cervical softening as preceding shortening in the first trimester, supporting SWE’s utility for early labor prediction [16]. Finally, Hu et al. proposed a quantitative system combining stress measurement with ultrasound, providing consistent elasticity metrics and extending the clinical applicability of cervical elastography [17].

### 2.2. Predictive Value of Cervical Elastography for Preterm Birth

Assessing cervical integrity and competence is essential for predicting preterm birth (PTB). Quantitative strain elastography evaluates factors such as reduced cervical stiffness, shorter cervical lengths, and increased endocervical canal width, which are strongly linked to PTB risk. Elevated strain values at the internal os are a measurable marker of cervical softness, a critical factor in premature cervical ripening. Combined with markers such as uterocervical angle and cervical length, elastography significantly enhances PTB prediction accuracy, allowing clinicians to identify at-risk pregnancies earlier and enable timely interventions to improve outcomes.

It should be noted that the etiology of PTB is highly heterogeneous, as highlighted in recent conceptual work advocating for a functional taxonomy that categorizes PTB into specific phenotypes based on etiological and clinical factors [18]. This classification framework underscores that cervical length, while an important marker, should not be considered in isolation. Instead, integrating multi-marker approaches, such as combining cervical length with immune-mediated factors or placental biomarkers, could improve the detection and risk stratification of spontaneous PTB [19]. Within this context, cervical elastography offers a complementary tool that could further refine risk prediction by providing dynamic, real-time assessments of cervical biomechanics, thus enhancing the overall predictive accuracy in multi-marker screening models.

A foundational study in 2014 demonstrated that semi-quantitative SE could distinguish changes in cervical stiffness, showing that elevated strain values correlated significantly with PTB. In this study, a strain threshold was identified, where values exceeding 0.89 were associated with a higher likelihood of PTB, with a specificity of 86% and a moderate sensitivity of 59% [20]. This finding supported the value of elastography as a PTB predictor and motivated further research to refine and expand its clinical application. This foundational study laid the groundwork for subsequent research.

Subsequent studies highlighted the value of elastography in both high-risk and low-risk pregnancies. For patients with short cervical lengths, qualitative SE assessments at the internal os indicated that softer cervical tissue predicted higher PTB risk. Sensitivity and specificity reached 82.2% and 75%, respectively, suggesting that elastography, when used alongside cervical length, provides a more robust prediction model than cervical length alone [21]. For low-risk pregnancies, quantitative SE using the E-Cervix software was shown to improve prediction capabilities, particularly during the second trimester. The area under the curve (AUC) for elastographic measurements at the internal os in this group was 0.730, underscoring elastography’s role in identifying potential PTB in cases where traditional risk factors may not be evident [22].

Expanding on previous research, Natarajan et al. incorporated multiple parameters in a recent SE study, including cervical length, anterior uterocervical angle, and qualitative cervical strain color maps. This integrative approach showed that combining cervical strain with other parameters enhanced the predictive accuracy of PTB, capturing risk factors that might be missed if only a single parameter, such as cervical length, were used [23]. Another SE study underscored the reliability of cervical strain ratios, with findings indicating that higher strain at the internal and external os corresponded with increased PTB likelihood [7]. This study supported the notion that cervical softness, as quantified by strain ratios, could serve as an early indicator of premature cervical ripening, a key contributor to PTB.

In addition, a recent study by Nazzaro et al. explored the utility of the E-Cervix system, a quantitative SE tool, for predicting preterm birth in singleton pregnancies with threatened PTL [24]. Their findings revealed that women who delivered preterm had significantly lower hardness ratios (HR), particularly with HR thresholds of <50% and <35%, compared to those who delivered at term. The study demonstrated that E-Cervix offers a sensitivity of 76% and specificity of 85% for HR < 50% and provides superior predictive accuracy when combined with cervical length measurements obtained via transvaginal ultrasound. This highlights the potential of strain elastography in improving preterm birth prediction, particularly in clinical settings where accurate risk stratification is critical.

**Figure 1:**
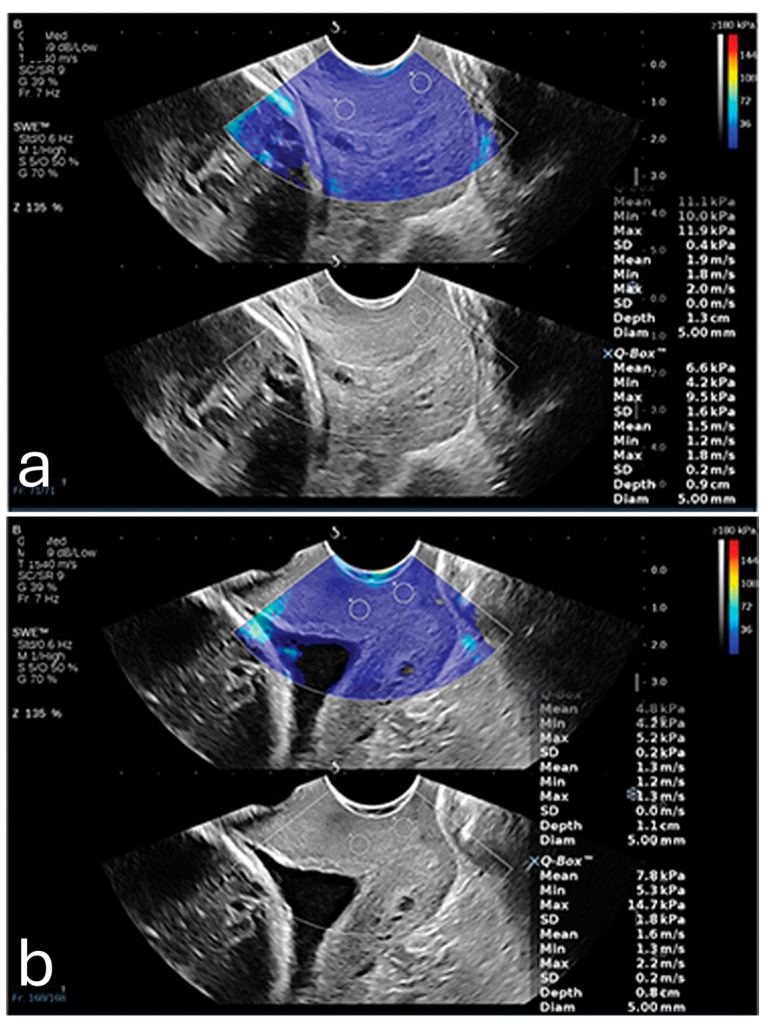
(**a**) Shear wave elastography image from a 26-year-old woman at 35 weeks gestation, showing high stiffness (11.1 kPa) in the anterior internal os; this patient delivered naturally at 38 + 4 weeks. (**b**) Shear wave elastography image from a 31-year-old woman at 34 weeks gestation, showing reduced stiffness (4.8 kPa) in the anterior internal os; this patient experienced preterm birth at 35 + 2 weeks due to premature rupture of membranes. Image adapted from Smith et al. [25] under a Creative Commons Attribution 4.0 International License. Changes were made to resize and adjust the images.

Furthermore, a 2024 study demonstrated that SWE could precisely measure tissue elasticity at specific cervical points, such as the inner and outer cervical os, yielding predictive values higher than cervical length alone [25]. When incorporated into a multifactorial model that included cervical length and pregnancy-related comorbidities, SWE achieved an AUC of 0.892, with a sensitivity of 86.7% and specificity of 79.2%. It also showed that SWE values were lower in PTB (8.76 kPa ± 3.04 kPa) in comparison to full-term pregnancies (14.95 kPa ± 8.21 kPa). These findings were most consistent when samples were taken from the anterior or posterior lip of the internal os, as illustrated in Figure 1. This combined approach shows promise for developing a more comprehensive PTB risk model that integrates both elastographic data and clinical factors [25].

Several studies underscore the promise of sonoelastography in assessing cervical insufficiency and competence [26,27,28,29,30]. Chen et al. found that semi-quantitative strain elastography (SE) in the first trimester revealed higher strain rates in the anterior cervical lip of women with prior cervical insufficiency, highlighting its potential for early risk identification [30]. Shorter cervical lengths and wider endocervical canals were also key predictors. Mlodawski et al. demonstrated the good repeatability and reproducibility of quantitative SE using the ‘E-Cervix’ software in the third trimester, emphasizing its value for longitudinal assessments [29]. Qu et al.’s systematic review and meta-analysis further validated SWE as a reliable tool for measuring cervical stiffness, showing consistent results in anterior and posterior cervical lips despite anatomical variances [31]. Collectively, these findings highlight the utility of elastography in enhancing early detection and intervention strategies for cervical competence.

### 2.3. Predicting Successful Labor Induction

In predicting labor induction success, studies on cervical elastography reveal mixed results on its utility and accuracy compared to traditional assessment tools like the Bishop score and cervical length. The Bishop score, while widely utilized for assessing cervical readiness for labor induction, has notable limitations. Its reliance on subjective clinical assessment often leads to variability in scoring between observers, reducing its reproducibility. Recent analyses suggest that even modified Bishop scores fail to account for the complexity of cervical remodeling processes and lack a direct correlation with physiological markers of labor readiness [32]. This subjectivity may result in inconsistent predictions, particularly in nulliparous women and cases of medically indicated preterm labor. Furthermore, as Kuba et al. discuss, modern induction methods and patient demographics differ significantly from those studied during the development of the Bishop score, further limiting its applicability in contemporary obstetric practice [32].

Pereira et al. assessed the potential of semi-quantitative SE scoring and the angle of progression (AOP) as predictors for labor induction outcomes [33]. They found significant correlations between cervical length, AOP, and elastography; however, only cervical length and parity proved to be strong predictors of vaginal delivery success and induction-to-delivery intervals. This study suggests that elastographic scores, specifically at the internal os, may have limited predictive power compared to cervical length, which remained a primary independent predictor of successful induction outcomes.

Mlodawski et al. evaluated the SE E-Cervix system for labor induction, developing a multifactorial ultrasonographic model to represent the Bishop score. The study demonstrated good repeatability and reproducibility for certain parameters but identified weak correlations between clinical cervical softness, as assessed by the Bishop score, and elastographic measures such as internal os strain (IOS) and external os strain (EOS), reflecting limitations in SE consistency or inherent flaws in the subjective Bishop score [34]. Similarly, Fruscalzo et al. found that quantitative SE modestly predicted labor induction failure but was not significantly more accurate than the Bishop score or cervical length [35]. These findings suggest that while elastography can enhance labor readiness assessments, it remains an adjunctive tool requiring refinement to improve its predictive reliability.

Londero et al. conducted a systematic review and meta-analysis comparing cervical elastography, cervical length, and the Bishop score [36]. The analysis revealed that both cervical elastography and cervical length had a higher diagnostic odds ratio (DOR) for predicting successful vaginal delivery compared to the Bishop score, suggesting that elastography provides a similar level of predictive reliability as cervical length. This reinforces the notion that elastography can be a valuable, objective measure, particularly in cases where cervical length alone is insufficient.

Lu et al. demonstrated that inner cervical SWE values were independent predictors of overall cesarean section likelihood and specifically predicted cases where labor failed to reach the active phase [37]. Models incorporating SWE and cervical length yielded significantly higher predictive accuracy than models based solely on the Bishop score, marking SWE as a promising technique for enhancing induction success assessments.

These findings collectively suggest that while cervical elastography may not replace existing methods like cervical length and the Bishop score, it has value as an adjunct tool, particularly in cases where objective cervical stiffness measurements could provide further insights.

## 3. Placental Assessment

### 3.1. Placental Elastic Properties

The placenta’s biomechanical properties, specifically its elasticity, are rooted in its unique anatomical and physiological structure, which evolves throughout gestation to support fetal development [38]. The elasticity of placental tissue largely reflects the organization and integrity of its core structural components, including the chorionic villi and the extensive vascular network. These structures enable nutrient and gas exchange, requiring flexibility to meet the fetus’s growing demands [39].

**Figure 2:**
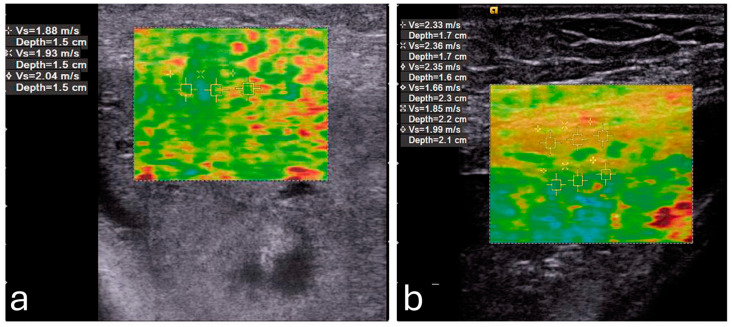
A pregnant woman with placenta percreta showing (**a**) increased average shear wave velocity (1.95 m/s) in comparison to a pregnant woman with normal placenta showing (**b**) normal average shear wave velocity (1.83 m/s). Reproduced with permission from Sage. © 2024.

One of the primary factors influencing placental elasticity is the layered arrangement of the chorionic villi, which are densely populated with capillaries and responsible for mediating maternal–fetal nutrient transfer. As trophoblastic cells invade and remodel maternal spiral arteries early in pregnancy, the placental vascular network expands, allowing the tissue to maintain elasticity and accommodate increased blood flow. These vascular adaptations support fetal growth and contribute to the biomechanical softness measured by elastography [40]. In contrast, elastography detects increased stiffness in cases of placenta percreta, as illustrated in Figure 2 [41].

Research shows a natural decline in placental elasticity as gestation advances, reflecting vascular and stromal maturation. Studies highlight that this softening results from the structural evolution of the placental tissue, wherein the density and arrangement of the villi and blood vessels continue to shift to optimize efficiency [40]. This progressive change is essential for maintaining placental functionality, as it ensures adequate resilience and flexibility to sustain fetal growth and health.

In normal pregnancies, these elastic properties are well-regulated, enabling a balance between structural stability and the adaptability necessary for dynamic blood flow adjustments. By understanding the baseline elasticity in healthy placental tissue, deviations observed in elastographic measurements can be more accurately interpreted, providing valuable insight into potential complications such as preeclampsia or intrauterine growth restriction [42].

### 3.2. Placental Adhesion Spectrum Assessment

Beyond general elasticity properties, sonoelastography provides specific insights into conditions like the placental adhesion spectrum (PAS), which encompasses conditions like placenta accreta, increta, and percreta, represents a significant challenge in modern obstetrics due to its associated maternal and fetal morbidity and mortality. PAS is characterized by abnormal placental implantation and invasion into the myometrium and beyond. Early detection is critical for optimizing patient outcomes through timely multidisciplinary management.

Emerging evidence highlights the potential role of sonoelastography in the evaluation of PAS. A study by Cim et al. used Virtual Touch Quantification (VTQ) techniques, a relatively older generation of elastography, to assess placental invasion anomalies [34]. They found significantly higher shear wave velocities (SWV) in cases with detected invasion compared to non-invasive cases across all regions of the placenta, with an average SWV of 2.862 ± 0.815 m/s in the invasion group versus 0.926 ± 0.484 m/s in the non-invasion group (*p* < 0.001) [34].

Dokumaci and Uyanikoglu further supported these findings by demonstrating that SWE could differentiate placenta percreta cases from normal pregnancies [41]. In their case-controlled study, the mean SWV values of the maternal edge of the placenta were 1.95 ± 0.19 m/s for placenta percreta cases and 1.69 ± 0.23 m/s for controls, with a cutoff value of 1.92 m/s, achieving 80% specificity for detecting PAS [41].

Additionally, Davutoglu et al. observed similar increases in placental stiffness in invasive placental conditions using SWE [43]. Their results suggest that elastography may reflect underlying histopathological changes such as infarction, inflammation, and trophoblastic invasion.

These studies collectively indicate that sonoelastography is a promising, non-invasive modality for assessing PAS. While current findings are encouraging, larger multicenter studies are required to establish standardized elastographic thresholds and protocols for PAS diagnosis. Such advancements could refine early diagnostic capabilities and improve the management of this high-risk obstetric condition.

### 3.3. Predicting and Monitoring Preeclampsia

Multiple studies demonstrate that placental stiffness values are significantly higher in pregnancies complicated by preeclampsia, with predictive power noted from the first trimester [38,42,44,45,46].

Killic et al. were one of the first researchers who found that SWE measurements could effectively distinguish between preeclamptic and normal placentas, reporting a significant increase in stiffness across all regions in preeclamptic cases [38]. The highest differences appeared at the fetal-facing central placental region, where SWE values reached a median of 21 kPa in preeclamptic cases versus 4 kPa in controls. The authors identified a diagnostic cutoff of 7.35 kPa, achieving 88% sensitivity and specificity, suggesting SWE’s utility in differentiating at-risk cases as early as the second trimester.

Cimsit et al. found no statistically significant variations in the elastic modulus values between areas, although they did find connections concerning increases in the placental modulus values of respective PE groups [4]. Later, Fujita et al. conducted a study to evaluate whether SWE could serve as a marker in the first trimester [42]. They found that SWE measurements in high-risk patients were significantly elevated, with an optimal cutoff of 1.188 m/s for detecting preeclampsia risk. This study highlighted that increased placental stiffness might correlate with early pathophysiological changes associated with the condition, supporting SWE’s potential for early detection in routine screenings.

Further support for SWE’s use in early detection comes from Sirinoglu et al. [46], who explored SWE in low-risk pregnancies during the first trimester. They identified an SWE cutoff of 7.43 kPa, achieving 88% sensitivity and 78% specificity, suggesting that SWE can reliably predict preeclampsia even in the absence of traditional risk factors. This study suggests that SWE might be advantageous as a primary screening tool, reducing reliance on other biochemical markers that require lab processing.

Building on SWE’s diagnostic capability, recent studies explore combined approaches for enhanced accuracy, such as a recent study by Tian et al., where SWE was combined with 3-dimensional power Doppler indices (3D-PDI) to test a potential improved predictive accuracy [44]. They found that combining SWE with vascularization indices (e.g., flow index and vascularization flow index) improved the sensitivity and specificity for preeclampsia prediction compared to SWE alone. Another recent study by Singh et al. assessed SWE in high-risk pregnancies at mid-gestation, revealing significantly higher elasticity values in preeclamptic placentas compared to controls, especially at the central and peripheral placental regions [45]. Their study suggested a diagnostic threshold of 13.1 kPa, achieving a high sensitivity of 95.2% and specificity of 92.8%, which indicates SWE’s potential as an adjunctive screening measure for high-risk pregnancies.

### 3.4. Detecting Fetal Growth Restriction

For detecting fetal growth restriction (FGR) through placental elasticity, few studies showcased sonoelastography as an emerging, promising approach [47,48,49]. Each contributes nuanced insights into placental stiffness as a marker for FGR, emphasizing elasticity differences between FGR and healthy pregnancies and the correlation with perinatal outcomes.

Habibi et al. utilized SWE to assess in vivo placental elasticity, identifying significantly higher stiffness values in FGR cases. This increase in placental stiffness—recorded at both central and peripheral regions—correlated with adverse outcomes, including lower birth weights and Apgar scores [48]. This study highlights SWE’s diagnostic capacity, suggesting its role as a supplementary tool to traditional Doppler measures, offering quantitative insights into placental health linked to FGR.

Akbas et al. conduced a case-control study where they revealed a distinct stiffness increase in FGR cases versus healthy controls, with stiffness values showing a strong correlation with Doppler indices and perinatal risks [49]. Notably, this study underscores that elevated placental elasticity values align with compromised placental function, reinforcing the value of SWE as a potential early diagnostic tool for FGR when standard ultrasound measures might miss early-stage abnormalities.

In a broader recent study, Ansar et al. examined SWE’s utility not only for detecting FGR but also for staging its severity, noting progressive stiffness elevations from mild to severe cases [47]. This study reinforces that increased stiffness mirrors worsening placental pathology, suggesting that SWE may be capable of staging FGR and enabling tailored monitoring for higher-risk pregnancies. This study showed that SWE could offer a more direct assessment of placental tissue alterations rather than indirect signs alone.

## 4. Pelvic Floor Muscles Assessment

### 4.1. Elastic Properties of the Pelvic Floor Muscles

The pelvic floor muscles (PFM), particularly the levator ani muscle (LAM) and the external anal sphincter (EAS), are essential for maintaining pelvic support and enabling control over movement within the pelvic region. These muscles exhibit a unique combination of elasticity and viscoelasticity, which allows them to withstand and adapt to various physiological forces, including those exerted during pregnancy, childbirth, and daily physical activities [50].

**Figure 3:**
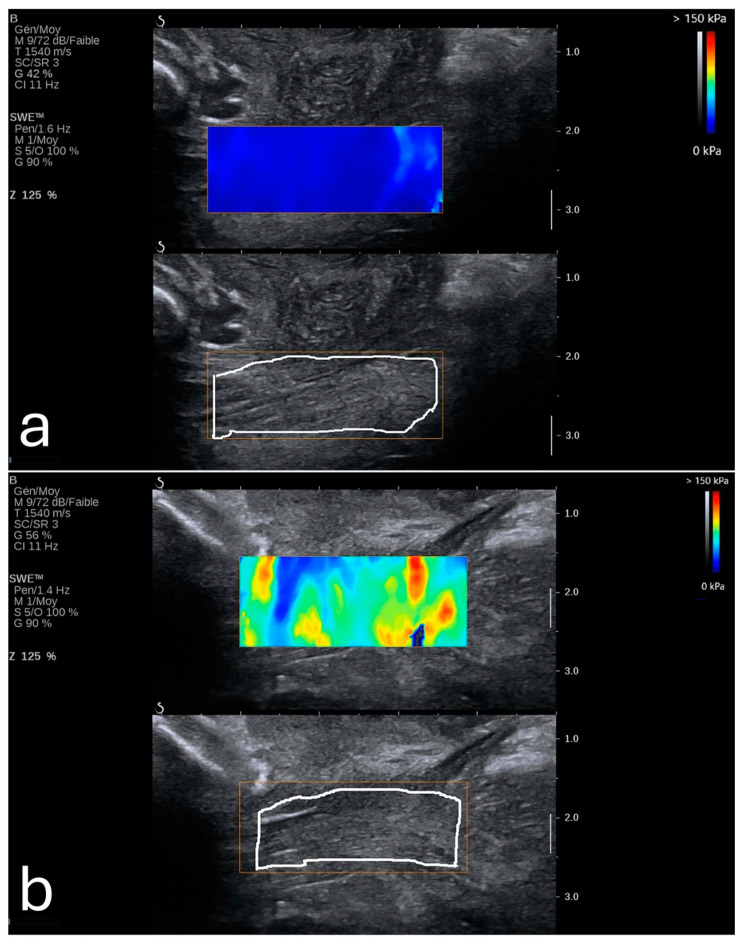
Shear wave elastography of the levator ani muscle at rest (**a**) and during a Valsalva maneuver (**b**). Image adapted from Gachon et al. [56] under a Creative Commons Attribution 4.0 International License. Changes were made to resize and adjust the images.

Elasticity in the PFM enables them to respond dynamically to increased abdominal pressure and other physical stresses. For instance, during actions such as the Valsalva maneuver or maximal contraction, the LAM and EAS demonstrate considerable elasticity, which provides resilience against deformation (Figure 3) [51]. This quality is particularly critical for maintaining the integrity of the pelvic organs and facilitating controlled movement.

The viscoelastic properties of these muscles also play a crucial role. Viscoelasticity allows the muscles to exhibit both solid-like and fluid-like behavior under stress, meaning they can absorb and dissipate energy effectively. This dual response is vital for protecting the pelvic structures from injury and supporting the body’s functional demands over time [52]. The PFM’s ability to adjust stiffness levels across different states—such as resting, contracting, or stretching—also highlights their adaptability, which is influenced by factors like hormonal changes during pregnancy and biomechanical demands [53].

It is crucial, however, to note that examining muscles using sonoelastography should be performed using the correct technique due to the anisotropic nature of these muscles [54]. In such instances, the probe should be oriented along the muscle fibers to ensure correct dissipation of the transverse shear waves [55].

### 4.2. Clinical Applications

Assessing PFM elasticity, particularly in the LAM and EAS, is relevant to prenatal diagnosis due to its critical role during pregnancy and childbirth. Sonoelastography can help predict childbirth-related injuries, such as perineal tears and anal sphincter injuries, guiding delivery strategies, including labor interventions or cesarean delivery, to improve maternal outcomes. Additionally, evaluating PFM stiffness during pregnancy may identify women at risk for postpartum complications, such as prolapse or incontinence, enabling timely rehabilitation and enhancing recovery.

A key application of PFM elasticity assessment lies in predicting childbirth-related injuries. Research has shown that increased stiffness in the EAS is associated with a lower risk of perineal tears and obstetric anal sphincter injuries during vaginal delivery. For instance, Gachon et al. conducted a study indicating that women with a stiffer EAS in late pregnancy had a reduced likelihood of experiencing perineal tears [57]. This finding suggests that SWE measurements can serve as early indicators of trauma risk, guiding clinicians in implementing preventative strategies or opting for modified delivery management techniques to reduce injury [57,58].

Postpartum recovery assessment is another significant application of SWE in clinical practice. Okada et al. demonstrated that elasticity in the LAM differed significantly between women who underwent vaginal delivery and those who had cesarean sections [58]. Their study revealed that postpartum women who delivered vaginally exhibited lower LAM elasticity, with an average elasticity difference of 28.2 kPa (*p* = 0.0036), potentially indicating muscle relaxation or injury. This measurable reduction in elasticity serves as a reliable metric for evaluating postpartum pelvic health and identifying cases where pelvic floor rehabilitation may be warranted to prevent conditions like pelvic organ prolapse and urinary incontinence [59].

For patients with symptoms of pelvic floor dysfunction, including incontinence and prolapse, SWE assessments provided an objective means of quantifying muscle elasticity loss, often a characteristic of such disorders. Davidson et al., for example, found that the active force and stiffness of the LAM varied significantly during pregnancy and postpartum (*p* = 0.002) [60]. This highlights SWE’s usefulness in monitoring muscle function over time. Such tailored rehabilitation may accelerate recovery and improve the efficacy of physical therapy interventions for those with pelvic floor dysfunction.

SWE’s capability extends to monitoring PFM functionality across various conditions. The technology allows elasticity to be measured under different states—such as at rest, during Valsalva, or under maximal contraction—providing a comprehensive view of muscle adaptability. This feature is particularly valuable in cases where gradual muscle weakening or functional impairments are suspected, allowing clinicians to track changes in muscle strength and elasticity that might necessitate early intervention.

In addition to its practical applications, SWE-based assessments of PFM elasticity are valuable in research, advancing our understanding of pelvic floor biomechanics. Studies on elasticity provide a foundational understanding of how pelvic floor muscle characteristics impact obstetric outcomes, inform prevention strategies, and support therapeutic approaches in maternal health. Davidson et al.’s work contributes to this body of knowledge, showing that active force and elasticity measurements could enhance the understanding of how PFM resilience is impacted by the demands of pregnancy and childbirth [61].

## 5. Fetal Assessment

**Figure 4:**
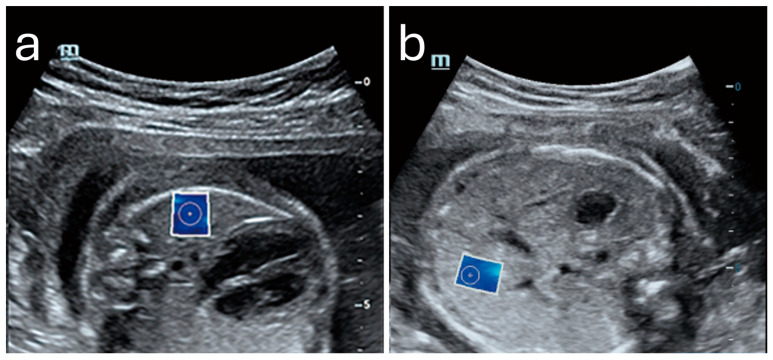
Normal shear wave elastography images of fetal lung (**a**) and liver (**b**) with elasticity values of 4.43 kPa and 5.09 kPa, respectively. Image adapted from Liu et al. [67] under a Creative Commons Attribution 4.0 International License. Changes were made to resize and adjust the images.

Sonoelastography offers a unique window into fetal development by providing quantitative insights into tissue properties and markers for identifying developmental progress and complications. By measuring the elastic properties of key organs such as the lungs, liver, and brain, clinicians could potentially evaluate organ maturity, detect potential anomalies, and make informed decisions regarding perinatal care [3,62,63,64,65,66,67,68,69]. Figure 4 shows an example of a normal lung and liver in a fetus.

### 5.1. Lung Elasticity and Maturity Assessment

SWE-measured fetal lung stiffness correlates with maturity markers and helps assess neonatal respiratory distress syndrome (RDS) risk. Research by Nallet et al. has documented that lung stiffness typically increases from the second trimester, peaking around 32 weeks of gestation, which corresponds with the development of alveolar structures and the production of surfactant necessary for respiratory function [62]. Beyond 32 weeks, lung elasticity values tend to decrease slightly, which may indicate that the tissue is reaching the final stages of maturation in preparation for the demands of postnatal breathing [62,63]. The typical values of lung stiffness range from around 4 kPa in early gestation to slightly over 5 kPa at their peak, then tapering as full-term approaches [3].

An important metric derived from fetal elastography is the lung-to-liver elasticity (LLE) ratio, which has emerged as a non-invasive indicator of lung maturity [3,67]. This ratio, remaining consistently between 0.8 and 0.9 in healthy pregnancies, allows clinicians to contrast lung development with the more gradually maturing liver. The LLE ratio could especially be helpful in cases where premature delivery is a concern [67].

### 5.2. Liver Elasticity as a Developmental Benchmark

In addition to lung development, sonoelastography also provides insights into liver elasticity as a developmental benchmark. The fetal liver’s elasticity shows a steady, linear increase throughout gestation, reflecting its progressive functional and metabolic maturation. Unlike the lung, which exhibits peak and subsequent reduction in stiffness, liver elasticity increases consistently, typically ranging from about 3.86 kPa at 24 weeks to approximately 4.45 kPa by 39 weeks of gestation [62]. This gradual increase aligns with the liver’s role in metabolic preparation for postnatal life and is used as a reference value when examining the lung-to-liver elasticity ratio [3,67].

The stability and predictable progression of liver elasticity also offer a reliable baseline against which abnormalities in other tissues, particularly the lungs, can be detected. By comparing liver stiffness to lung stiffness, clinicians gain insight into whether lung development is progressing within expected limits, which is particularly useful in assessing fetuses at risk of developmental delays or other complications [62].

### 5.3. Brain Elasticity and Structural Maturation

Beyond lung and liver assessments, brain elasticity represents another critical area of sonoelastography applications. The brain has specific regions, such as the cerebral parenchyma and choroid plexus showing elasticity changes that correlate with brain development stages. Studies, including those by Zheng et al. reveal that elasticity in cerebral regions increases with gestational age, likely reflecting the progressive formation of neural structures and the complexity of brain tissue [68]. The elasticity of the cerebral parenchyma, for instance, tends to be higher than other regions like the choroid plexus, suggesting its dense cellular organization and critical role in neural activity.

Clinical applications of brain elasticity assessment extend to detecting structural abnormalities and monitoring development in high-risk pregnancies. In one case study exploring the elasticity of a fetal brain affected by atypical choroid plexus papilloma, the elasticity values provided additional information on the lesion’s characteristics, aiding in diagnosis and management [69]. While these clinical applications are promising, understanding the safety of SWE in fetal assessments is paramount.

### 5.4. Safety in Fetal Assessment

The safety of fetal sonoelastography, specifically SWE, has been investigated due to concerns over the potential bioeffects of the high-intensity acoustic radiation force used to generate shear waves. The primary areas of concern are thermal effects, mechanical impacts on tissue, and possible teratogenicity.

Thermal effects, due to the absorption of acoustic energy, are measured by the thermal index (TI). Studies such as those conducted by Issaoui et al. [64] suggest that SWE’s thermal impact is like that of pulsed Doppler ultrasound, which is widely accepted in obstetrics. However, SWE involves brief energy peaks that could lead to localized heating, especially at bone-tissue interfaces, where thermal buildup might approach safety thresholds.

Mechanical effects include tissue displacement caused by the acoustic radiation force, measured by the mechanical index (MI). Although no direct cavitation or significant displacement effects have been documented in clinical settings, in silico studies raise caution about potential impacts on delicate fetal structures, such as the cochlea, which may be vulnerable to high-intensity shear vibrations. Preliminary findings by Massó et al. [70] in newborns showed no increase in hypoacusis or adverse birth outcomes following prenatal SWE, though further research is recommended to substantiate these results across larger populations [70,71].

Overall, current evidence supports SWE as safe within regulated TI and MI limits, but the findings highlight a need for cautious application, adhering to ALARA (As Low As Reasonably Achievable) principles to mitigate potential risks associated with acoustic peaks. Further studies are encouraged to expand understanding of SWE’s long-term bioeffects on fetal tissues.

## 6. Conclusions

Sonoelastography offers significant promise in prenatal diagnostics by providing a non-invasive method to assess tissue elasticity and stiffness in maternal and fetal structures. Its applications span cervical assessments for preterm birth prediction, placental evaluations for conditions like preeclampsia and intrauterine growth restriction, and fetal organ assessments to monitor development and detect anomalies. These capabilities enhance the diagnostic power of traditional ultrasound, offering earlier and more precise insights into maternal and fetal health.

Current evidence supports the safety of sonoelastography within established guidelines. Further research is needed to standardize protocols, confirm long-term safety, and expand clinical applications. As this technology continues to evolve, it holds the potential to become a cornerstone in prenatal care, improving outcomes through more informed and timely interventions.

## 7. Future Directions

As the utility of sonoelastography in prenatal diagnosis grows, key future directions include developing standardized protocols and gestational age-specific reference ranges for tissue elasticity to improve diagnostic precision and reliability. Advancements in shear wave elastography (SWE) could enhance image resolution and reduce artifacts, enabling better assessment of complex anatomical regions like the cervix and fetal organs. Machine learning integration holds promise for automating elasticity assessments and improving diagnostic accuracy and efficiency.

Long-term safety studies on SWE are crucial to evaluate its impact on fetal development and newborn outcomes, ensuring adherence to safety guidelines. Combining sonoelastography with other diagnostic tools, such as Doppler ultrasound or biochemical markers, could provide comprehensive risk assessments. For example, elasticity metrics paired with vascular indices in placental evaluation or cervical biomarkers in preterm birth prediction could enhance personalized care. Collaboration between radiology and obstetrics will be essential to refine protocols and expand its clinical applications, positioning sonoelastography as a cornerstone in prenatal diagnostics.
